# Dysfunction of the oligodendrocytes in amyotrophic lateral sclerosis

**DOI:** 10.7555/JBR.36.20220009

**Published:** 2022-08-28

**Authors:** Zhenxiang Gong, Li Ba, Min Zhang

**Affiliations:** Department of Neurology and Psychiatry, Tongji Hospital, Tongji Medical College, Huazhong University of Science and Technology, Wuhan, Hubei 430030, China

**Keywords:** oligodendrocytes, amyotrophic lateral sclerosis, energy metabolism, oxidative stress, neuroinflammation

## Abstract

Amyotrophic lateral sclerosis (ALS) is a fatal neurodegenerative disorder characterized by irreversible deterioration of upper and lower motor neurons (MNs). Previously, studies on the involvement of glial cells in the pathogenic process of ALS have mainly revolved around astrocytes and microglia. And oligodendrocytes (OLs) have only recently been highlighted. Grey matter demyelination within the motor cortex and proliferation of the oligodendrocyte precursor cells (OPCs) was observed in ALS patients. The selective ablation of mutant SOD1 (the dysfunctional superoxide dismutase) from the oligodendrocyte progenitors after birth significantly delayed disease onset and prolonged the overall survival in ALS mice model (SOD1^G37R^). In this study, we review the several mechanisms of oligodendrocyte dysfunction involved in the pathological process of myelin damage and MNs death during ALS. Particularly, we examined the insufficient local energy supply from OLs to axons, impaired differentiation from OPCs into OLs mediated by oxidative stress damage, and inflammatory injury to the OLs. Since increasing evidence depicted that ALS is not a disease limited to MNs damage, exploring the mechanisms by which oligodendrocyte dysfunction is involved in MNs death would contribute to a more comprehensive understanding of ALS and identifying potential drug targets.

## Introduction

Amyotrophic lateral sclerosis (ALS) is a fatal neurodegenerative disease characterized by the degeneration of upper and lower motor neurons. Researchers have made great efforts to explore the pathological mechanism and therapies for ALS^[1]^. Unfortunately, the efficacy of the two currently approved drugs, *viz.* Riluzole and Edaravone, is limited^[2–3]^. Revealing the initiating and aggravating factors of neuronal degeneration is the first step toward developing new therapies. A significant body of evidence has shown that ALS is not strictly a disease of motor neurons (MNs)^[4–5]^. And there is a complex network of interactions between glia and MNs^[6–8]^. Many agents have shown preliminary efficacy on animal models based on the modulation of glial cells and are being investigated in clinical trials^[9]^. Dysfunction of the glial cells has become the focus of ALS research and a promising target for developing novel therapies.

Glial cells in the central nervous system (CNS) mainly include astrocytes, microglia, and oligodendrocytes (OLs). It is unclear whether the dysfunction of glial cells is either the cause or the consequence of neuronal degeneration in ALS. Previous studies confirmed the importance of neuron-surrounding cells in the pathology of ALS. The cell-specific expression of mutant superoxide dismutase1 (mSOD1) in MNs, astrocytes, or microglia did not cause ALS-like motor deficit. In addition, the selective reduction in mSOD1 expression within microglia and astrocytes does not prevent the onset of the disease but significantly slows the progression^[6,10–14]^. Therefore, it is inevitable that the aggregation of mutant mSOD1 in glial cells causes glial dysfunction, accelerating motor neuron injury and disease progression.

**Figure 1:**
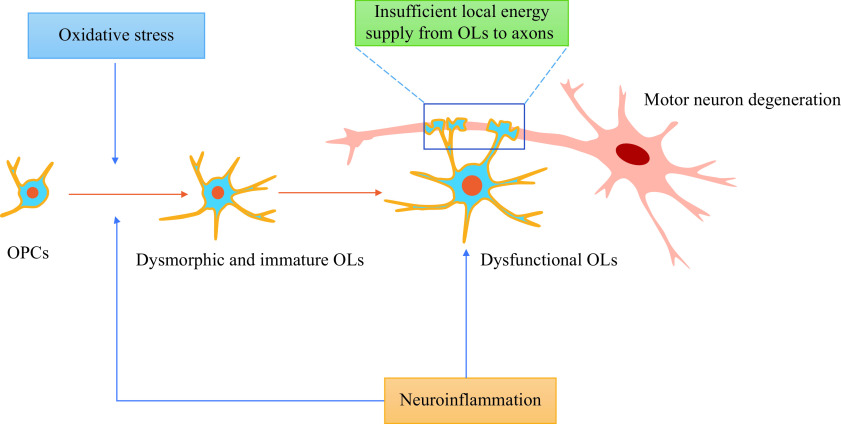
Oligodendrocyte dysfunction is involved in motor neuron death in amyotrophic lateral sclerosis.

Unlike astrocytes and microglia with active and resting forms, OLs have distinct morphological characteristics and physiological functions. They are the most numerous cells in CNS and are in close contact with the axons of MNs. The main physiological processes of OLs in maintaining and supporting motor neuron survival are as follows: (1) OLs are the essential cells that make up the sheath, ensuring axon integrity and rapid nerve impulse conduction^[15]^. When demyelination occurs, oligodendrocyte precursor cells (OPCs) differentiate into OLs under complex regulations to replace the apoptotic OLs^[16]^; (2) OLs provide trophic and metabolic support for neurons, especially local energy supply for axons^[8,17–18]^ because neurons are characterized by high energy expenditure, limited glycogen storage, and inability to acquire extracellular glucose; (3) OLs regulate immune networks between the glial cells and neurons^[19–20]^. Therefore, the dysfunction of OLs will directly or indirectly affect the degeneration of MNs (***Fig. 1***). Demyelination of motor neuron axons occurs in the spinal cords of SOD1^G93A^ mouse and grey matter and spinal cords in ALS patients^[21]^. In the mice model of ALS (SOD1^G93A^), degeneration of mature OLs in the gray matter of the spinal cord precedes visible symptoms and motor neuron death^[21–22]^. OPCs proliferate inside the spinal cord of SOD1^G93A^ mice at the pre-symptomatic stage but could not compensate for the loss of mature OLs^[23]^. Selective ablation of mutant SOD1 from OPCs (NG2^+^ cells) after birth significantly delayed the disease onset and prolonged the overall survival in ALS mice (SOD1^G37R^)^[21]^. Moreover, specific overexpression of mutant SOD1 in mature OLs induced demyelination, promoting the degeneration of MNs and resulting in an ALS-like cell phenotype in a zebrafish model^[24]^. Therefore, the evidence depicts that oligodendrocyte dysfunction is involved in the death of MNs.

Since ALS is not strictly a motor neuron disorder, pathological events between neurons and glial cells are significant. In the past decades, the involvement of glial cells in the pathogenic process of MNs death has revolved around astrocytes and microglia. However, the role of OLs and OPCs has been described in the pathogenic events occurring in ALS. Our objective was to review the mechanisms of oligodendrocyte dysfunction involved in the pathological process of myelin damage and motor neuron death in ALS. The study will deepen the knowledge of the involvement of OLs in the pathological process of ALS, and shed light on potential drug targets.

## Dysfunction of local metabolic supply from oligodendrocytes to axons in ALS

Neurons are glucose-dependent for energy supply; however, the active transport of extracellular glucose coupled with oxidative phosphorylation mainly occurs in the neuronal cell body^[25]^. A hydrophobic barrier surrounds long axons formed by OLs in CNS and Schwann cells in the peripheral nervous system (PNS)^[26]^. For this reason, the energy deficit of neurons is expected in pathological situations, especially in neurodegenerative diseases, including ALS. When neurons are energetically deprived, energy metabolites are indirectly taken from the surrounding cells to ascertain biochemical activities, especially OLs^[27]^. The blocked pathway of OLs supplying energy to axons could be involved in the degeneration of MNs in ALS.

### Glucose shuttling

Oligodendrocyte networks can predominantly provide energy to sustain the axonal function through glucose delivery^[28]^. This indirect manner of energy supply is named glucose shuttling by some researchers. In the brain, the corpus callosum is a structure in which the number of OLs is much greater than that of astrocytes and microglia. Meyer *et al* depicted that exogenous glucose deprivation (EGD) led to abolishing compound action potentials (CAPs) in the acute slices of the mouse corpus callosum. However, loading an oligodendrocyte with 20 mmol/L glucose prevented EGD-mediated CAP reduction, demonstrating the glucose delivery from OLs to axons^[28]^. The glucose transport can rely on the connexin (Cx) families, which develop intact connexin gap junctions and diffuse the small molecules, including glucose. In the human brain, connexin 32 (Cx32) and Cx47 are predominantly expressed on the membrane surface of OLs, while Cx30 and Cx43 are expressed on the membrane surface of astrocytes^[29]^. Interestingly, a previous study found that Cx47 and Cx32 on the membrane surface of OLs markedly decreased in the anterior horns of spinal cords of the transgenic mice model of ALS, indicating a potential dysfunction of intercellular trafficking of glucose from OLs to MNs^[30]^. Because glucose is the most important energy substance directly available to MNs, impaired shuttling of glucose from OLs to MNs can lead to a state of energy deficiency in MNs.

### Lactate shuttling

The most important substance for non-neuronal cells to supply energy to neurons is not glucose, but lactate. In neurodegenerative diseases, there was a substantial anaerobic glycolysis response in glial cells for compensating abnormalities in energy production which transforms glucose into lactate in the cytoplasm. Many previous studies confirmed that astrocytes could supply lactate to neurons without glucose^[31–32]^. Recently, OLs are likely to have similar functions of providing lactate to neurons. In the optic nerve glucose deprivation experiment, researchers observed that the N-methyl-D-aspartic acid (NMDA) receptor on the membrane surface of OLs was activated to enhance the trafficking of glucose into OLs by the glucose transporter 1, thus increasing anaerobic glycolysis and facilitating the downstream transfer of lactate from OLs to axons^[33]^. Another essential monocarboxylate transporter 1 (MCT1), a vital lactate transporter, was identified in the myelin sheath around CNS axons^[34]^. In both the ventral spinal cord of the transgenic mice model and the motor cortex of ALS patients, OLs were deficient in MCT1^[35]^. Insufficient expression of MCT1 on the membrane of OLs can aggravate the energy deficit of MNs in ALS. The impairment of lactate shuttling from the OLs to the MNs could exacerbate the degeneration of MNs in an energy-dependent manner in ALS.

### Metabolic crosstalk between astrocytes, oligodendrocytes, and neurons

Astrocytes play a vital role in the energy support of neuronal cells. In resting periods, astrocytes take up an equivalent amount of glucose as neurons. Additionally, astrocytes can actively reduce fatty acids into ketone bodies as metabolic nutrients, and pyruvate (a kind of ketone body) can be efficiently utilized by neurons during glucose deficiency, establishing astrocytes as the formidable provider of alternative energy metabolites^[36-37]^. Fibrillar astrocytes associate with both MNs and OLs at nodes of Ranvier, constituting a physiological and functional network. As previously described, heterotypic gap junctions between the astrocytes and OLs consisted of Cx32, Cx47, Cx26, Cx30, and Cx43, allowing OLs with astrocyte-derived metabolic nutrients. In humans, mutations in Cx32 cause X-linked Charcot-Marie-Tooth disease, and the mutations in Cx47 lead to Pelizaeus-Merzbacher-like disease^[38–39]^. Except for gap junctions, astrocytes can produce and release lactate, which could be picked up by OLs, into the extracellular space^[40]^. OLs may indirectly supply neurons with energy by acquiring energy substrates from the astrocytes-oligodendrocytes network under either pathogenic or energy-deprived conditions. Disruption of the metabolic energy network between glial cells may be involved in motor neuron death. And the metabolic crosstalk among neurons, astrocytes, and OLs requires further understanding and is an exciting frontier to be explored.

## Dysregulation of oligodendrocyte precursor cells differentiating into oligodendrocytes mediated by oxidative stress in amyotrophic lateral sclerosis

Reactive oxygen species and reactive nitrogen species are the two dominant pro-oxidants derived from mitochondria-related dysfunction. Oxidative stress is essential in accelerating neuronal degeneration in neurodegenerative diseases, particularly ALS^[41]^. The pharmacological target of Edaravone is to scavenge excessive oxygen-derived free radicals in the MNs of ALS patients^[42]^. OPCs are most proliferative in glial cells of the CNS^[43–44]^. OPCs continuously differentiate into OLs, repairing the demyelination of axons. The extremely high proliferation process promotes the differentiation of OPCs under increased energy demand conditions. Active mitochondrial-related biochemical processes elevate reactive oxygen species production in OPCs, and lead OPCs to be more vulnerable to oxidative stress damage, eventually accelerating the degeneration of MNs.

In previous studies, neurons, astrocytes, and microglia have been mainly elaborated on mitochondrial dysfunction and oxidative stress damage. Some preliminary experiments revealed that oxidative stress could destroy OLs, cause dysfunction of OPCs, and demyelinate the grey matter in both the motor cortex and spinal cord in ALS^[21–22]^. OPCs are proliferated in the spinal cord of mSOD1^G93A^ mice during the pre-symptomatic stage^[23]^. However, they cannot differentiate into mature OLs, leading to irreversible demyelination and axonal degeneration during ALS. Little is known about the process of oxidative stress damage hindering the differentiation of OPCs into OLs in ALS. According to previous experiments, there were two possible mechanisms leading to the dysregulation of differentiation from OPC cells into the OLs in ALS.

First, the endogenous defense mechanism of OPCs and OLs against oxidative stress is relatively vulnerable in ALS patients, attributed to the mSOD1 deposition. On the one hand, SOD1 is vital for converting active oxygen ions to H_2_O_2_^[45]^. However, mSOD1 in OLs loses this ability, resulting in excessive accumulation of intracellular active oxygen in the OLs. The upregulation of SOD1 with normal physiological functions reduced the oxidative stress-mediated death in OPCs and promoted the differentiation of OPCs to mature OLs^[46–47]^. On the other hand, an essential antioxidant protection strategy is inducing the expression of nuclear factor erythroid 2-related factor 2 (Nrf2), a main eukaryotic redox-active factor from the basic leucine zipper transcription factors. Nrf2 can bind to the promoter regions of antioxidant response element (ARE), activating genes that encode antioxidant proteins, including the Thioredoxin reductase 1, the NAD (P)H-quinone oxidoreductase 1, and the Heme oxygenase-1 (HO-1)^[48]^. *In vivo*, actin-bound cytoskeletal zinc metalloprotein Kelch-like ECH-associated protein1 (Keap1) is an Nrf2 inhibitor, which binds to Nrf2 and subsequently degrades the ubiquitin-proteasome system^[49]^. Decreased binding of Nrf2-Keap1 will result in the intranuclear shuttling of Nrf2 and subsequent transcription of the antioxidant proteins. However, there was an increased Keap1 mRNA expression in the motor cortex in postmortem samples of patients with ALS^[50]^. In multiple sclerosis (MS), selective deficiency of Nrf2 increases the OLs loss, demyelination, and axonal damage^[51]^. However, no study has specifically demonstrated the changes of Keap1or Nrf2 in OLs of ALS. Recently, a significant decrease in Nrf2 and HO-1 in brain homogenate was found in a rat model of ALS. With the oral supplementation of Acetyl-11-keto-beta-boswellic acid, a multi-component pentacyclic triterpenoid mixture, and an Nrf2/HO-1 activator, the myelin basic protein (MBP) increased in the brain homogenate, suggesting a potential role of Nrf2/HO-1 mediated signaling pathway of MBP restoration in ALS^[52]^.

Secondly, oxidative stress can affect DNA damage in both OLs and OPCs, causing the inability of OPCs to differentiate into the mature OLs. The linkage between oxidative stress and DNA damage in OLs has been adequately illustrated in MS^[53–55]^. In the previous studies of MS, high levels of 8-hydroxyguanine, the most common DNA oxidative lesion, were primarily observed in oligodendrocyte nuclei^[55]^. In addition, the pathological association between oxidative damage and oligodendrocyte destruction is supported by the evidence from immunocytochemistry for malondialdehyde (MDA) and oxidized phospholipids^[55–56]^. Moreover, OLs and OPCs appear to have a weaker ability to repair DNA damage than astrocytes and microglia. When exposed to oxidative stress due to menadione, OLs were more susceptible to mitochondrial DNA (mtDNA) harm than the astrocytes. The decreased mtDNA repair positively correlated with the enhanced susceptibility toward apoptosis in OLs^[57]^. The regeneration of myelin sheath requires the continuous differentiation and supplement of OPCs. High levels of oxidative stress in ALS could hinder the differentiation of OPCs due to the lack of efficient DNA repair, resulting in irreversible axonal degeneration of MNs. Therefore, future research needs to focus on the linkage between oxidative stress damage and the OLs in ALS.

## Neuroinflammation injury in oligodendrocytes and oligodendrocyte precursor cells

Previous studies have demonstrated that neuroinflammation affects the function of OLs, leading to abnormal demyelination and remyelination in many diseases, including classic demyelination, ischemic stroke, and neurodegenerative disorders^[58]^. At the same time, OLs are not just the victims of neuroinflammation but also have the potential to act on the inflammatory process. On the one hand, inflammation factors impact OLs and OPCs. Proinflammatory cytokines, such as tumor necrosis factor (TNF), lead to OPC cell damage and prevent the differentiation from OPCs to mature OLs^[59–60]^. In addition, interleukin-1β (IL-1β) could induce OLs apoptosis and hypomyelination^[61]^. Interferon gamma (IFN-γ) can also influence the major histocompatibility complex class Ⅰ antigen presentation pathway and results in OPC death^[62]^. On the other hand, OLs can sense the inflammation and react to it *via* expressing cytokines, inflammasomes, and chemokines, including TNF-α, IL-1β, IL-17, IFN-γ, CCL2, CXCL10, and CXCR1, which along with astrocytes and microglia, allows OLs to participate in the neuroinflammatory network^[19]^. In addition, astrocytes could transport the inflammation-related small molecules from the blood to OLs using the gap junctions consisting of connexins. Moreover, astrocytes and microglia secrete proinflammatory cytokines that create an abnormal immune microenvironment, damaging OLs by the mechanisms mentioned above. Conversely, OPCs maintain the quiescent state of microglia and control the intensity of microglia activation^[63–64]^. In the MPTP-induced PD mouse model, the deficiency of OPCs exacerbates the activation of microglia and hence, induces dopaminergic neuron loss^[64]^.

Neuroinflammation is a common feature of many neurodegenerative diseases, including ALS. Evidence has revealed that high levels of neuroinflammation play a vital role in the pathogenesis and progression of ALS^[65–67]^. Moreover, peripheral immune activation has also been shown in ALS models and patients with changes in peripheral blood cells and cytokines, such as reduced Treg cells and elevated IL6. However, inflammation is a double-edged sword. Degradation of damaged myelin debris and regeneration requires appropriate inflammatory stimulation^[68]^. However, a high-level inflammatory immune microenvironment would hinder myelin regeneration and promote the degeneration of OL and MNs. More in-depth investigations are required to explore the link between neuroinflammation, OLs, and motor neuron death in ALS.

## Conclusions

Although many efforts have been made to understand the pathological mechanism involving ALS, no effective therapies are available. Moreover, attention was primarily paid to astrocytes and microglia in the past decades. However, recent studies have shown an important role for oligodendroglial cells in the pathological process involving ALS. Insufficient local energy supply from OLs to axons, impaired differentiation from OPCs to OLs mediated by oxidative stress, and a proinflammatory immune microenvironment could cause axon impairment, thus, accelerating the death of neurons and overall disease progression. Additionally, as a disease involving motor and non-motor neuron cells, a more comprehensive understanding of the glia-neuron networks could help develop new therapies that delay disease progression.
